# Deaths of profound despair: A retrospective cohort study of mortality among people experiencing homelessness

**DOI:** 10.1371/journal.pone.0281912

**Published:** 2023-02-16

**Authors:** Jamie Suki Chang, Katherine Saxton, Georgia Bright, Michelle A. Jorden, Andy Gutierrez, Katherine Xia

**Affiliations:** 1 Department of Public Health, Santa Clara University, Santa Clara, California, United States of America; 2 Office of the Medical Examiner-Coroner, County of Santa Clara, San Jose, California, United States of America; 3 Office of the Public Defender, Post-Conviction Outreach Unit, County of Santa Clara, San Jose, California, United States of America; King’s College London, UNITED KINGDOM

## Abstract

**Introduction:**

The number of people dying while unhoused is increasing nationally. In Santa Clara County (SCC), deaths of unhoused people have almost tripled in 9 years. This is a retrospective cohort study examining mortality trends among unhoused people in SCC. The objective of the study is to characterize mortality outcomes in the unhoused population, and compare these to the SCC general population.

**Materials and methods:**

We obtained data from the SCC Medical Examiner-Coroner’s Office on unhoused people’s deaths that occurred between 2011–2019. We analyzed demographic trends and cause of death, compared to mortality data on the SCC general population obtained from CDC databases. We also compared rates of *deaths of despair*.

**Results:**

There were a total of 974 unhoused deaths in the SCC cohort. The unadjusted mortality rate among unhoused people is higher than the general population, and unhoused mortality has increased over time. The standardized mortality ratio for unhoused people is 3.8, compared to the general population in SCC. The most frequent age of death among unhoused people was between 55–64 years old (31.3%), followed by 45–54 (27.5%), compared to 85+ in the general population (38.3%). Over ninety percent of deaths in the general population were due to illness. In contrast, 38.2% of unhoused deaths were due to substance use, 32.0% illness, 19.0% injury, 4.2% homicide, and 4.1% suicide. The proportion of deaths of despair was 9-fold higher in the unhoused cohort compared to the housed cohort.

**Discussion:**

Homelessness has profound impacts on health, as people who are unhoused are dying 20 years younger, with higher rates of injurious, treatable, and preventable causes, than people in the general population. System-level, inter-agency interventions are needed. Local governments need to systematically collect housing status at death to monitor mortality patterns among unhoused people, and adapt public health systems to prevent rising unhoused deaths.

## Introduction

Home is the essential place where health is established, and health is in jeopardy without one. Disparities in mortality rates and causes among people experiencing homelessness have been reported throughout the United States and globally [1–4]. According to national estimates, there are nearly 600,000 people who are homeless (i.e. unhoused, houseless) in the United States [5]. Homelessness is defined as individuals and families living in a supervised publicly or privately operated shelter designated to provide temporary living arrangement; or with a primary nighttime residence that is a public or private place not designed for or ordinarily used as a regular sleeping accommodation for human beings, including a car, park, abandoned building, bus or train station, airport, or camping ground [6].

In recent years, public health practitioners and journalists across the United States reported sharp increases in numbers of people dying while homeless [7]. By 2018, fourteen cities published reports on deaths of unhoused people, and in almost all cities, the number of deaths notably increased in recent years [7]. While mortality rates and causes varied by region, reports consistently showed that unhoused people had greater risk of death, substantially lower life expectancy, and that men and people of color were at highest risk [7]. Current estimates of unhoused mortality are almost certainly undercounts because in many cities or counties, housing status is not available at time of death, nor is reporting of homelessness required on death records [7].

The County of Santa Clara (SCC), California, USA—known as Silicon Valley—has a large unhoused population, and notably, has among the highest proportions of unhoused-unsheltered people in the US [8]. The 2019 unhoused point-in-time count (PITC) estimated that approximately 9,700 unhoused people live in SCC, with four out of five (82%) living unsheltered [9]. In SCC, the rise in unhoused deaths was first reported by the Medical Examiner-Coroner’s office (MEC) in 2016 [10]. They found that the number of people dying while unhoused more than doubled between 2011–2016. Since this report was published, the number of deaths has continued increasing almost every year, spiking from 162 deaths in 2019, to 203 deaths in 2020 [11].

The number of deaths of unhoused people is increasing, but epidemiologic research characterizing unhoused mortality is limited. Public health groups have called for more systematic data collection, as currently there is no federal count of unhoused deaths [7]. Most academic studies have focused on cause of death (COD) or risk factors for early death at the city or county level [12]. Factors involved in unhoused deaths include being unsheltered and having substance use issues [13]. Most deaths of unhoused people involve men, and there are gender differences in mortality risk factors [14]. Veterans, older adults, youths aged 15–25, and children under 18 who are unhoused are at greater risk of mortality than their housed counterparts [15–18]. Although the primary CODs vary by region, they often include deaths related to substance use, injuries, and illnesses including cancer and heart disease [7, 12, 19].

Most research in homeless mortality reports on individual-level characteristics because this type of data is available in MEC reports. To shift the focus to more upstream, structural causes, we borrow from the work on *deaths of despair (DOD)*, a term that characterizes patterns of death linked to hopelessness and decreasing socioeconomic status, job opportunities, and social capital. This term initially characterized declining mortality rates observed between 1999–2013 among middle-aged, white communities, with less than a high school education [20, 21]. The concept of deaths of despair is debated. Researchers have criticized how it centers “white despair” and disregards or misrepresents mortality trends in other racial/ethnic groups [22]. More recent studies have suggested that mortality rates have also begun accelerating among people of color [23]. Researchers also caution that the concept is limited due to its individualized, deficit focus [24, 25]. Despite these criticisms, DOD is a useful concept that underscores how systemic, structural factors such as socioeconomic decline can affect population health, including mortality outcomes [23, 26].

DOD is an evolving concept, as researchers debate the parameters of what causes of death can be considered as a death of despair. The majority of the literature has reached consensus that the specific CODs that comprise DODs are suicide, chronic liver disease, and drug and alcohol poisoning. Researchers have noted, however, that increased rates of other chronic diseases such as cancer, cardiovascular disease, respiratory disease, diabetes, and obesity may also contribute to DOD mortality rates [23, 27].

There is a dearth of epidemiological research characterizing mortality of unhoused people in general, and none in SCC. The intention of this study was to describe patterns in mortality in the unhoused population, compared to the general population in SCC, and to apply the concept of DOD to this population to explore the relationship between structural conditions and cause of death. We hypothesized that compared to the general population in SCC, unhoused people 1) have higher mortality rates, 2) die at younger ages, and are more likely to be male, 3) die of different causes of death, and 4) have higher rates of DOD.

## Materials and methods

### Study design, setting, and participants

We performed a retrospective cohort study, comparing mortality between unhoused people and the general population living in SCC for 2011–2019. For the unhoused cohort, we obtained mortality data for all people who died and were identified as unhoused in SCC between January 1 2011 to December 31 2019 from the MEC office. For a deceased individual to be included in the unhoused cohort, they had to be identified as being homeless by the SCC MEC. Only cases that were reviewed by the MEC office count toward the official homeless death count in SCC. The MEC office identified a deceased individual as unhoused if they were either living on the street or if they had no valid living address at the time of death. Once identified as unhoused, homeless status was confirmed based on a medico-legal investigation, which included examining the circumstances and environment attending a death, interviewing people who knew the deceased individual, and verifying with next-of-kin. All unhoused descendants underwent an autopsy to determine COD. For the general population cohort, we used the CDC WONDER Underlying Cause of Death database to identify all deaths in SCC for 2011–2019 and collected data on COD, age, sex, and year of death.

### Ethics statement

This study was approved by the Institutional Review Board in the Office of Research Integrity and Compliance at Santa Clara University. The data used in this study for both cohorts is publicly available, and no written or verbal consent was obtained.

### Variables and data sources

Variables that we examined between each cohort included: year of death, age, sex, and the primary COD.

**Fig 1:**
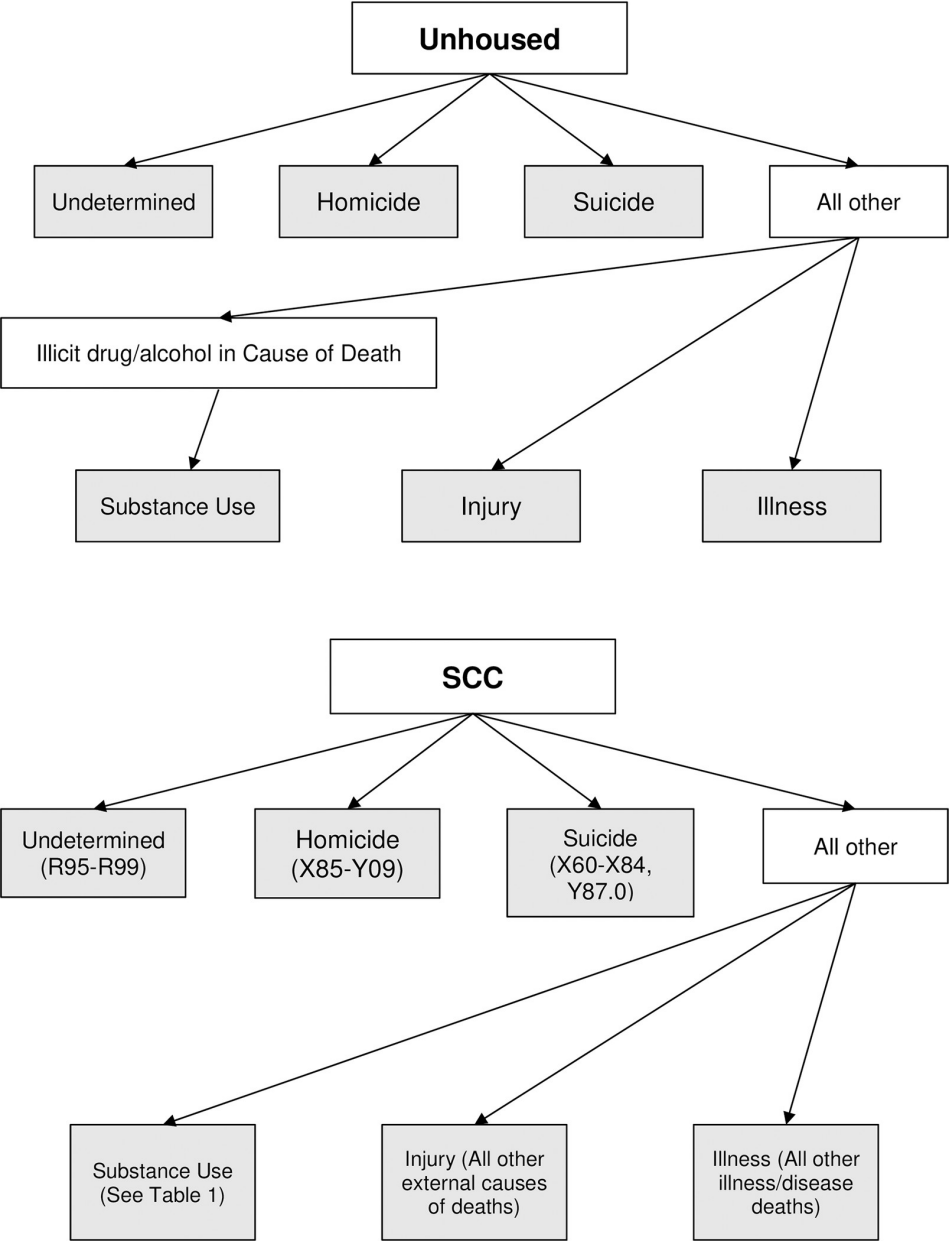
Categorization of causes of death for the unhoused cohort and the Santa Clara County general population.

For the unhoused cohort, the MEC office maintains a database, including an open data portal (https://medicalexaminer.sccgov.org/medical-examiner-coroner-dashboard), that compiles and summarizes data from unhoused people’s autopsy reports, including year of death, age, sex, and the primary COD. Cause of death was reported as clinical descriptions on the death certificate, rather than International Statistical Classification of Diseases (ICD) codes. We used COD clinical descriptions to create the following primary, mutually exclusive categories: Homicide, Suicide, Undertermined, Substance use, Injury, and Illness (1–6) (Fig 1).

1 *Homicide*: Determined by the Medical Examiner.
2 *Suicide*: Determined by the Medical Examiner.
3 *Undetermined*: Cause of death could not be determined or was unavailable.
4 *Substance use*: Cases involving one or more drugs and/or alcohol, including “natural” deaths (e.g. cirrhosis) and “accidental” deaths (e.g. overdose/poisoning, drug-related injury). If more than one substance was mentioned in the primary cause of death, it was coded as “Polysubstance Use”.
5 *Injury*: Cases attributed to intentional or unintentional injury (e.g. blunt force injury, falls).
6 *Illness*: Communicable and non-communicable illnesses.

We then compiled specific CODs to create the DOD category (7):

7 *Deaths of despair*: Cases involving suicide, substance use, as defined above, and liver disease.

For the general population cohort, the CDC COD data reports illness/disease and external causes. We assigned SCC deaths to the categories created for the unhoused population (Table 1). For Substance Use deaths, we included both acute poisonings and deaths related to chronic substance use [19, 28]. We also assigned specific CODs to match the theory-based categories (Table 1).

**Table 1:** Categorization of ICD-10 codes for the Santa Clara County general population cause of death between 2011–2019. Categories 1–6 are primary categories that were identified from CDC WONDER Underlying Causes of Death database. Category 7 is constructed based on the deaths of despair framework.

		CDC External Causes	CDC Illness/Disease
1	Homicide	X85-Y09	--
2	Suicide	X60-X84, Y87.0	--
3	Undetermined		R95-R99
4	Substance Use	X40-45, Y10-15, Y45, 47, 49	E24.4, F10, F11–F16, F18–F19, G31.2, G62.1, G72.1, I42.6, K29.2, K70, K85.2, K86.0, O35.4, O35.5, P96.1, Q86.0
5	Injury	All other external cause deaths	
6	Illness	--	All other illness/disease deaths
***Deaths of Despair***			
7	Deaths of Despair	Suicide: X60-84, Y87.0	Chronic Liver Disease: K70, K73-74
		Drug and Alcohol Poisoning: X40-45, Y10-15, Y45, 47, 49	

### Statistical analysis

We used the SCC point-in-time-count (PITC) to estimate the size, age, and sex distribution of the unhoused population. The PITC is a survey of unhoused people typically conducted on a single night in January every other year [29]. During this survey, both sheltered and unsheltered individuals are counted. We then compared mortality rates of the unhoused and general populations by calculating deaths per 100,000 population for each year of the study period. We calculated mortality rate ratios and 95% confidence intervals. We examined changes in unhoused death over time using linear regression for the number of deaths in each population and the proportion of unhoused people’s deaths.

We used data from the CDC WONDER Underlying Cause of Death database to calculate the average age at death for the general population. We found a weighted average using the count of deaths that occurred at each age over the study period (2011–2019). We used a goodness of fit test to compare the age distribution of deaths in the two populations (age ranges: <1, 1–14, 15–24, 25–34, 35–44, 45–54, 55–64, 65–74, and 85+ years). We combined age groups 1–4 and 5–14 for analysis, because of low numbers of deaths in the unhoused population (there were no unhoused deaths in the 5–14 year age group). Among the unhoused population, we used linear regression to examine the average age of death over time, stratified by sex. In each population, we used linear regression to examine the proportion of deaths of men vs. women over time.

We compared age-specific mortality rates for the unhoused and the general populations in SCC for 2019. Because the age and sex distribution of the unhoused population differs from the SCC general population, we adjusted for age and sex using indirect standardization methods. We applied the age-specific rates of death from the SCC general population in 2019 to the unhoused population size to calculate the number of expected unhoused deaths, if the unhoused cohort experienced the mortality rates of the general population. We then calculated standardized mortality ratios (SMRs) by taking the ratio of the observed deaths to the expected deaths. We repeated this process to adjust for sex. The PITC does not report the sex by age distribution of the unhoused population, so we were unable to adjust for sex and age together. We also calculated unadjusted mortality rate ratios (MRRs) to compare age- and sex-specific mortality.

We compared the frequency of COD categories for the unhoused and general populations by calculating the percent of deaths, and 95% confidence intervals, in each category (illness, injury, homicide, suicide, substance use, and unknown). We repeated this comparison for deaths categorized as DOD.

All analyses were performed using Stata 15 (College Station, TX) and Microsoft Excel.

## Results

The MEC identified a total of 974 deaths between 2011–2019 (Table 2). During the same time period, the CDC WONDER Underlying Cause of Death database identified a total of 88,658 deaths in SCC. The number of deaths of unhoused people increased 2.7 fold, from 60 in 2011 to 162 in 2019 (linear regression: F_1,7_ = 88.59, B_year_ = 13.17, p<0.001). During the same period, the biennial PITC indicates a 37% increase in the population of unhoused people in SCC (7,067 in 2011 to 9,706 in 2019).

**Table 2:** Characteristics of unhoused individuals who died in Santa Clara County between 2011 and 2019 (n = 974).

Characteristic	Total
	(n = 974)
**Sex**	**n (%)**
Male	796 (81.72%)
Female	164 (16.84%)
Unknown	14 (1.44%)
**Age**	**n (%)**
Younger than 18	3 (0.31%)
18–24	19 (1.95%)
25–30	35 (3.59%)
31–40	100 (10.27%)
41–50	200 (20.53%)
51–60	321 (32.96%)
61+	282 (28.95%)
Unknown	14 (1.44%)
**Year of Death**	**n (%)**
2011	60 (6.16%)
2012	69 (7.08%)
2013	87 (8.93%)
2014	79 (8.11%)
2015	97 (9.96%)
2016	138 (14.17%)
2017	142 (14.58%)
2018	140 (14.37%)
2019	162 (16.63%)

### Mortality rates

The unadjusted mortality rate among unhoused people is higher than the general population, and unhoused mortality has increased over time (linear regression: F_1, 3_ = 13.10, B_year_ = 121.02, p = 0.036). The unhoused mortality rate increased 197% from 849.0 deaths per 100,000 population (95%CI: 648.5, 1092.5) in 2011 to 1669.1 deaths per 100,000 (95% CI: 1423.6, 1944.1) in 2019. Meanwhile, the crude mortality rate among the general population in SCC has increased by only 4%, from 506.9 deaths per 100,000 (95% CI: 496.6, 517.3) in 2011 to 527.8 deaths per 100,000 (95% CI: 517.6, 538.1) in 2019 (linear regression: F_1,7_ = 28.01, B_year_ = 3.13, p = 0.001).

### Age and sex

#### Age

The mean age of death for the unhoused population was 53.3 years (95% CI: 52.5, 54.2), considerably lower than SCC population average age of death of 75.9 years (95% CI: 75.8, 76.1). Overall, unhoused people are more likely to die during middle age (45–54 and 55–64 years) than the general population (goodness of fit, df = 9, n = 963, X^2^ = 2325.4, p<0.001). We used indirect standardization to account for differing age distributions of the unhoused and general populations, using the PITC to estimate the size and age distribution of the unhoused population in 2019. The SMR (standardized mortality ratio) for unhoused people is 3.8, adjusting for age, compared to the general population in SCC.

Age-specific mortality differed between the unhoused and general populations, especially in early adulthood and middle age (Table 3). For example, in 2019, unhoused people aged 25–30 (age-specific mortality rate of 1717.2 per 100,000) were 40.1 times more likely to die (95% CI: 20.9, 76.9) compared to people of the same age in the general population (age-specific mortality rate of 42.87 per 100,000). The risk of death was increased nearly 15-fold for unhoused people aged 31–40 (MRR 14.9, 95% CI: 9.0, 24.7). However, older unhoused people, over age 60, died at twice the rate of the general population (MRR 1.9, 95%CI: 1.5, 2.5).

**Table 3:** Age-specific mortality rates for the Santa Clara County (SCC) unhoused population and the SCC general population (n = 974 deaths; 88,658 deaths respectively) and age-specific mortality rate ratios in 2019.

Age categories (Point-in-Time Count)	Number of unhoused deaths	Unhoused mortality rate (deaths per 100,000)	SCC mortality rate (deaths per 100,000)	Age-specific MRR (95%CI)^a^
<18	0	0.0	26.4	-
18–24	4	274.7	38.5	7.1 (2.6, 19.6)
25–30	10	1717.2	42.9	40.1 (20.9, 76.9)
31–40	16	1030.3	69.3	14.9 (9.0, 24.7)
41–50	32	1498.6	133.6	11.2 (7.8, 16.1)
51–60	42	1545.4	351.0	4.4 (3.2, 6.0)
61+	54	4636.3	2419.2	1.9 (1.5, 2.5)
Not stated	4	-	-	-
Total	162	1669.1	527.7	3.2 (2.7, 3.7)

^a^MRR = mortality rate ratio = unhoused mortality rate/SCC mortality rate

Some have suggested that the homeless population is getting older, and therefore the risk of death has increased over time because of this change in age structure. However, the age of death among unhoused people has not changed during the study period, neither for men (linear regression: F_1, 787_ = 2.64, p = 0.11) nor women (linear regression: F_1,160_ = 1.73, p = 0.19). The increasing age among unhoused individuals does not explain the increase in the number of deaths over the study period.

#### Sex

Men account for 50.9% (95%CI: 50.6%, 51.2%) of deaths in the general population in SCC. In the unhoused population, however, men account for 83% (95%CI: 80.4%, 85.2%) of deaths each year.

The SMR (standardized mortality ratio) for unhoused people is 3.2, adjusting for sex, compared to the general population in SCC. The unadjusted MRR is 3.16, which suggests that the increased mortality was not due to the sex-distribution of the unhoused population. The unhoused population includes a higher proportion of men than does the general population, and men experience higher mortality than women throughout adulthood. However, the elevated risk of death cannot be accounted for by the overrepresentation of men in the unhoused population.

Sex-specific death rates differed in the unhoused population and the general population. In 2019, unhoused men experienced a mortality rate 4.1 times higher (95% CI: 2.6, 6.5) than unhoused women. Unhoused men were also at increased risk of death, compared to the general population. Unhoused men (sex-specific mortality rate: 2346.9 deaths per 100,000) were 4.3 times (95% CI: 3.7, 5.1) more likely to die compared to men in the general population (sex-specific mortality rate: 542.9 deaths per 100,000). However, unhoused women (sex-specific mortality rate: 574.2 deaths per 100,000) were no more likely to die (MRR: 1.12, 95%CI: 0.72, 1.7) than women in the general population (sex-specific mortality rate: 512.3 deaths per 100,000).

### Cause of death

#### Substance use

**Fig 2:**
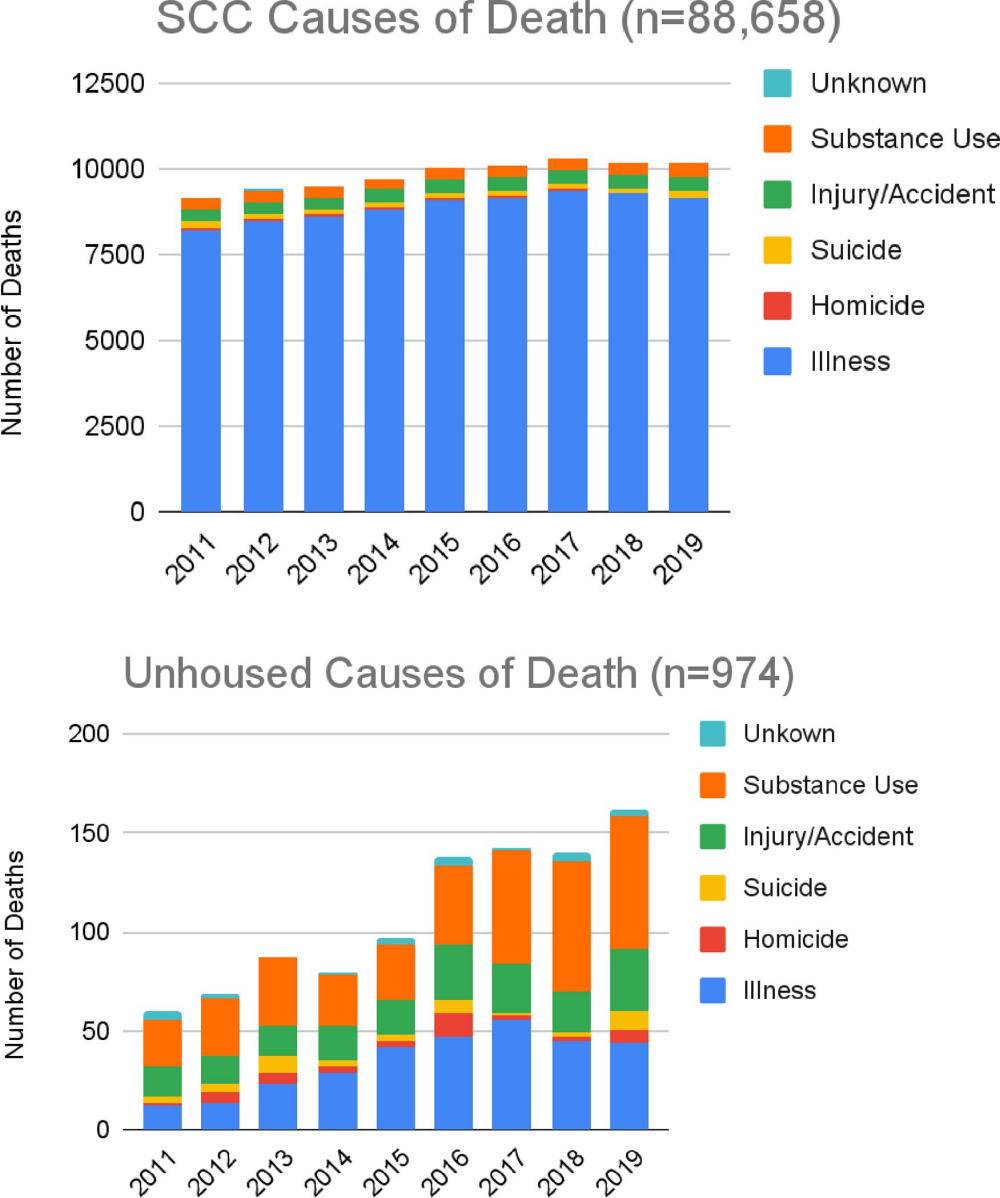
Number of deaths per year for the general population and the Santa Clara County (SCC) unhoused population between 2011–2019, displayed by cause of death categories.

In the general population, 3.3% of deaths were caused by substance use (95% CI: 3.2%, 3.4%), whereas 38.2% of unhoused deaths were due to substance use (95% CI: 35.1%, 41.3%) (Fig 2). In the unhoused population, most substance use deaths involved methamphetamine only (37%, n = 141), alcohol only (30%, n = 112), opioids only (3%, n = 12), and more than one substance (23%, n = 87). For unhoused deaths that involved more than one substance, 61% (n = 53) were due to a combination of opioids and methamphetamine (methamphetamine and opioids n = 36; methamphetamine, opioids, and other drugs, n = 8; methamphetamine, opioids, and alcohol, n = 4; methamphetamine, opioids, cocaine, n = 3; and methamphetamine, opioids, alcohol, and other drugs, n = 2).

#### Illness

In the general population, 90.4% of deaths were due to illness (95% CI: 90.2%, 90.6%), whereas 32.0% of unhoused people’s deaths were due to illness (95% CI: 29.1%, 35.1%). Among unhoused people’s deaths caused by illness, the top CODs were cardiovascular diseases (46.8%, n = 146), respiratory diseases (12.2%, n = 38), and cancer (8.3%, n = 26). Communicable diseases (4.8%, n = 15), diabetes (4.8%, n = 15), and cerebrovascular diseases (4.5%, n = 14) were notable.

#### Injury

In SCC, 4.0% (95% CI: 3.9%, 4.1%) of deaths were caused by injury. In the unhoused population, 19.0% (95% CI: 16.6%, 21.6%) of deaths were caused by injury. For unhoused people’s deaths caused by injury, most involved blunt force injury (46.5%, n = 86) and injury involving vehicles (19.5%, n = 36).

In the general population, 0.5% of deaths were caused by homicide (95% CI: 0.045%, 0.055%), and 1.6% suicide (95% CI: 1.5%, 1.7%). In the unhoused population, 4.2% of deaths were due to homicide (95% CI: 3.0%, 5.6%), and 4.1% suicide (95% CI: 2.9%, 5.6%).

### Deaths of despair

#### Deaths of despair

**Fig 3:**
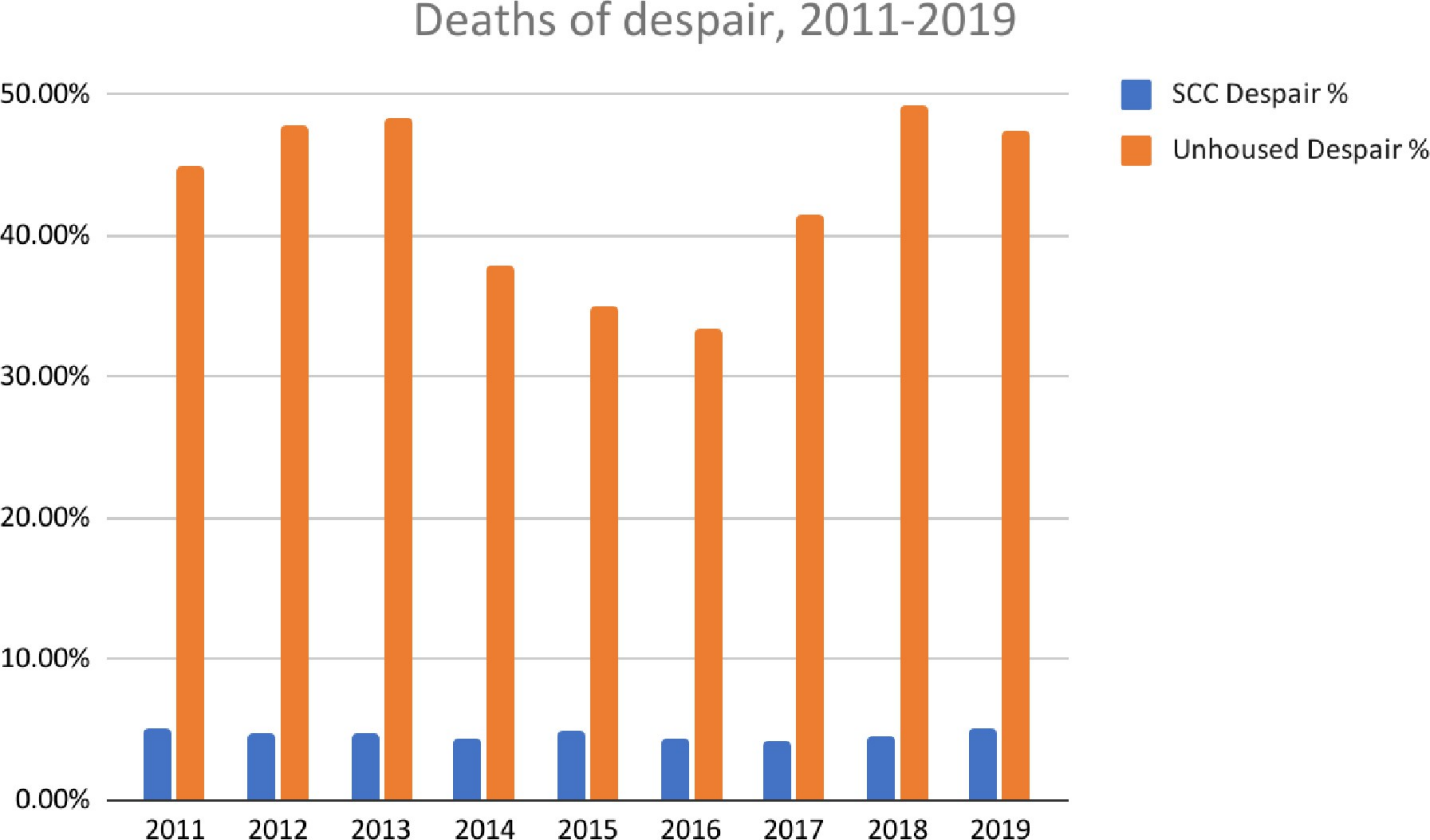
Proportions of deaths categorized as deaths of despair in the general population compared to the Santa Clara County (SCC) unhoused population between 2011–2019 (n = 88,658; 974 respectively).

In the general population, 4.7% (95% CI: 45.9%, 48.7) of deaths were DOD. Among unhoused people, in contrast, 42.8% (95% CI: 39.7%, 46.0%) of deaths were classified as DOD (n = 417), over nine times more than in the general population (RR = 9.1, 95% CI: 8.4, 9.8). In addition, the proportion of deaths considered DOD remained relatively constant among the general population, ranging from a low of 4.2% in 2017 to a high of 5.2% in 2019. However, DOD among the unhoused population were substantially higher throughout the study period, ranging from a low of 33.0% of deaths in 2016 to a high of 49.3% of deaths in 2018 (Fig 3).

## Discussion

Santa Clara County is one of the healthiest counties in the nation [30], but this county-wide metric hides stark health inequalities. The findings demonstrate major mortality disparities between people who are unhoused and housed. Deaths of unhoused people in SCC increased 2.7 fold between 2011–2019. The majority of unhoused people’s deaths occurred in middle age (58.8% between 45 and 64), compared to age 75 or over (59.74%) in the general population. Men accounted for most unhoused deaths, but increases in mortality rates were not attributable to proportions of men in the population. In the general population, most deaths (90.4%) were due to illness. In contrast, 38.2% of unhoused deaths were due to substance use, 32.0% illness, 19.0% injury, 4.2% homicide, and 4.1% suicide. Unhoused people experience DOD at nine times the rate of the general population.

Although inconsistencies in data limit regional comparisons, our findings are consistent with reports from other U.S. regions that show unhoused descendants were more commonly male and died in middle age. The overall SMR was 3.8, adjusting for age, compared to the general population of SCC. The majority of deaths in SCC occurred between 45–64, with an average age of 53.3, which is slightly older than other regions such as Sacramento, California, and Denver, Colorado, but younger than average ages reported in Seattle, Washington [7]. One finding that is worth noting is that in our study, unhoused women had no greater risk of death than women in the general population. This is in contrast to most studies that have shown unhoused women have increased risk of mortality compared to the general population [31, 32]. We are unable to determine the underlying cause of this contrast, which warrants further analysis.

This study has limitations. First, the sample size per year in the unhoused cohort—while tragically high—is relatively small for analysis and generalizability. A key limitation is that the CODs provided by the SCC MEC were clinical descriptions, which were interpreted to match with cause of death ICD codes used for the general population, and therefore the match is not identical. This study involved people who died while unhoused, whose cases were reviewed by the SCC MEC, and does not include cases that were not reviewed through this office. The findings describe mortality trends, but do not determine the scale of death that is directly attributable to being unhoused. Factors such as race, ethnicity, immigration status, and shelter status are key factors that we were unable to include in this analysis. The MEC data did not contain information on duration of homelessness, and the chronicity of homelessness is unknown. Santa Clara County has one of the highest costs of living in the country, with regionally-specific concerns on affordability and inequality, which limits generalizability.

These limitations notwithstanding, this study raises several implications. Foremost, there is a significant need for increased, improved systematic collection of unhoused mortality data at all governmental levels. Currently, there is no standardization or requirement to document housing status at the time of death, therefore most efforts to collect or analyze mortality data are done piecemeal at the municipal or county levels. There are no accurate national estimates, and many regions do not collect unhoused mortality data at all. Public health groups have developed recommendations for more widespread and systematic efforts toward collecting and reporting unhoused mortality data, such as gender, age, or race/ethnicity, but barriers exist such as de-centralized public health reporting systems in many states [7].

Housing status and mortality data can be combined to develop practical, localized public health intervention and implementation plans to address soaring death rates. The majority of unhoused deaths are preventable. Collecting data on housing status at the time of death enables public health departments to monitor patterns in unhoused deaths, and these data can be used to guide targeted system-level efforts to prevent unhoused deaths. For example, we found that a substantial number of unhoused people in SCC are dying from vehicle-involved injuries. This information can lead to prioritization of specific transit areas for increased pedestrian safety measures. We also found that a large number of unhoused people’s deaths involved drugs and alcohol, in particular methamphetamine. Most public health efforts toward addressing substance use have focused on opioids, but of all substances, methamphetamine use has the most devastating impact on unhoused people in the region. Prioritizing street-outreach-based methamphetamine treatment, developing pipelines for unhoused people to enter residential treatment, and bolstering methamphetamine treatment services in the shelter system are potential approaches for addressing methamphetamine-involved unhoused deaths.

We analyzed DOD to underscore the tragic circumstances surrounding unhoused deaths, and to emphasize the role of upstream, structural factors involved. Most studies examining unhoused deaths have focused on the individual level, but greater investigation into how structural arrangements shape the health risks and outcomes of individual unhoused people is needed. We encourage researchers to examine homelessness and homeless mortality using frameworks such as *structural vulnerability*, a justice-oriented framework used in health research for acknowledging, identifying, and rectifying the multiple forms of everyday risks built into the lives of people who are marginalized [33]. Interdisciplinary work shows how human-made social systems create disproportionate harm to groups of people, which is mediated by one’s status, position, and power within the social system. These structures are described as violent because they are consequential to health, often resulting in real injury or death [34]. The structural vulnerability perspective places structural conditions (such as homelessness and the lack of shelter) as upstream, fundamental causes of illness and disease for unhoused people, moving beyond a focus on individual factors (i.e. biological, pathological, behavioral) influencing poor health. Research into the effects of local, state, and national housing policies, criminalization of homelessness (e.g. encampment abatement policies), health delivery systems, etc. on mortality rates and other health measures can locate root-cause structural solutions.

## Conclusion

Homelessness is a major public health issue that has devastating health outcomes, as unhoused people face greater risk of mortality, die at younger ages, and of more injurious, treatable, and traumatic causes than the general population. As the numbers of deaths continue to soar every year, there is an urgent need for public health data, research, interventions, and resources to be deployed to reduce preventable suffering and untimely deaths of unhoused people.
